# Improvement of Vacuum Free Hybrid Photovoltaic Performance Based on a Well-Aligned ZnO Nanorod and WO_3_ as a Carrier Transport Layer

**DOI:** 10.3390/ma12091490

**Published:** 2019-05-08

**Authors:** Nguyen Tam Nguyen Truong, Hai Ha Thi Hoang, Chinho Park

**Affiliations:** School of Chemical Engineering, Yeungnam University, 280 Daehak-Ro, Gyeongsan 38541, Korea; tamnguyentn@ynu.ac.kr (N.T.N.T.); hahoangdna@gmail.com (H.H.T.H.)

**Keywords:** nanorods, well aligned, nanorods orientation, inclination angle

## Abstract

Well-aligned zinc oxide nanorods (WA-ZnO Nrods) with different lengths were synthesized and the effects of the growth times on the optical, morphological, and electrical properties of the WA-ZnO Nrods were examined. We also investigated the application of WA-ZnO Nrods as an electron transport layer (ETL) and tungsten trioxide (WO_3_) as a hole transport layer (HTL) to vacuum free hybrid photovoltaic (HPV) performance. The eutectic gallium–indium (EGaIn) alloy was used as a top electrode coated using a brush-painting method. A device with the structure of indium tin oxide (ITO)/WA-ZnO Nrods/(P3HT:PCBM)/WO_3_/EGaIn was optimized and fabricated. The maximum power conversion efficiency (PCE) was ~4.5%. Improvement of the device performance indicates that the well-aligned ZnO Nrods and WO_3_ can effectively be applied as charge carrier transport layer for vacuum free hybrid (HPV).

## 1. Introduction

Zinc oxide (ZnO) nanostructures have attracted considerable attention in both fundamental research and potential applications. ZnO materials have a wide band gap (3.34 eV) as well as a high exciton binding energy (60 meV) at room temperature, high electron mobility (100 cm^2^_V_^−1^ s^−1^), and piezoelectricity [1,2]. This relatively low-cost and environmentally friendly oxide semiconductor has applications in different fields, such as solar cells [3], transducers [4], sensors [5,6], photocatalysis [7], and UV lasers [8].

Controlling the size and shape of ZnO nanostructures is important for improving the properties and performance of solar cells [9,10]. One-dimensional (1-D) structures, such as nanorods, nanowires and nanotubes, are beneficial, because they offer a direct pathway for photo-generated electrons with fewer trapping photon sites [11,12]. The 1-D structure of ZnO can lead to improved rapid electron transport. Y. Hames [13] investigated the performance of organic solar cells based on ZnO nanorods and poly(3-hexylthiophene) (P3HT), and reported that a structure with ZnO nanorods has a higher power efficiency of 2.44% compared to that with ZnO nanoparticles (1.49%). They took advantage of the high electron transport ability of ZnO nanorods (Nrods) to fabricate organic solar cells on composite nanostructures of electron donor and acceptor materials as the electron-transport layer (ETL) [14,15,16]. In particular, the performance of organic solar cells (OSCs) made from ZnO Nrodpolymer blends showed improved electron mobility because of the infiltration of polymer into the gaps between the vertically aligned nanorods. In addition, the alignment of polymer chains along the rods and vertical structures provide large surface-to-volume ratios and high aspect ratios [17,18]. The hydrothermal method is a low cost, low temperature, and very simple method to synthesize well-aligned (WA)-ZnO Nrods [19,20,21]. The advantages of this method are easy control of the morphology and crystalline properties of the nanorod, as well as the coating of large area substrates without using either high vacuum or high-power sources. In order to archive good performance from an inorganic/organic HSCs, the introduction of an HTL film between the photoactive layer and the top metal electrode (like Silver (Ag)) is necessary. The most commonly materials used as HTLs are polymers (e.g., poly(3,4-ethylenedioxythiophene)-poly(styrenesulfonate) (PEDOT:PSS)) or metal oxides (e.g., molybdenum trioxide (MoO_3_), Divanadium trioxide (V_2_O_3_), Tungsten trioxide (WO_3_), Nickel(II) oxide (NiO)) [22]. They show that the best HTL for hybrid solar cells should be MoO_3_ and WO_3_ because of their ability to reduce oxygen, metal ions diffuse into the photoactive layer, improving of the hole collection. These results showed very good device efficiency in P3HT:PCBM based solar cells with a PCE exceeding 3%.

Eutectic gallium–Indium alloy has been applied as a cathode in the hybrid PVs configuration [23,24]. This alloy exists in the liquid phase at room temperature, has a melting point of ~15.5 °C, and is non-toxic. It can be deposited simply at the atmospheric environment and room temperature without need for the thermal evaporator technique or specialized equipment [25]. EGaIn also has high work function (~4.2 eV) similar to aluminum (~4.3 eV), and electrical conductivity (~3.4 × 10^4^ S cm^−1^) is very close to that of aluminum (~ 3.5 × 10^4^ S cm^−1^).

In this study, WA-ZnO Nrods were synthesized and applied to HPVs as an ETL and WO_3_ was used as a HTL layer. Devices with the structure of indium tin oxide (ITO)/electron transport layer (ETL)/(P3HT:PCBM)/hole transport layer (HTL)/EGaIn were fabricated and characterized. We controlled the morphology and electricity properties of carrier transport layer (ETL and HTL) to improve the device performance. The photoactive layer was prepared by mixing P3HT and C61-butyric acid methyl ester (PCBM). Eutectic gallium–Indium was used as a top electrode metal.

## 2. Materials and Methods

### 2.1. Chemicals

The reagents used throughout the experimental process were purchased from Sigma Aldrich (Seoul, Korea) and included zinc acetate dehydrate (Zn(Ac)_2_·2H_2_O, 99%), ethanolamine (MEA, NH_2_CH_2_CH_2_OH), 2-methoxyethanol (CH_3_OCH_2_CH_2_OH), zinc nitrate hexahydrate (Zn(NO)_3_·6H_2_O, > 99%), hexamethylenetetramine (HMTA; C_6_H_12_N_4_), P3HT, PCBM, EGaIn and WO_3_ nanoparticle ink. All chemical reagents were used without further purification. The patterned ITO/glass substrates were cleaned ultrasonically in isopropanol, acetone, and methanol for 15 min each, and then dried.

### 2.2. Preparation of the ZnO Seed Layer

A cleaned-ITO coated glass substrate was used as the substrate to grow the ZnO Nrods using sol-gel methods. Zinc acetate dehydrate (45 mM) and ethanolamine (45 mM) were dissolved in 2-methoxyethanol to prepare the precursor solution for the seed layer. The resulting solution was stirred for approximately 1 h at room temperature before being spin coated onto the ITO/glass substrate at 3000 rpm for 40 s, and annealed on a hot plate at 300 °C for 20 min to form the oriented crystalline ZnO seed layer (~30 nm).

### 2.3. Preparation of the WA-ZnO Nrods

The precursor solution for growing the ZnO Nrods was prepared by dissolving 60 mM Zn(NO)_3_·6H_2_O and 60 mM HMTA in deionized (DI) water in a beaker. The ITO/ZnO seed layer was immersed in a solution in a beaker and placed in a Teflon stainless steel autoclave at 90 °C. The size and length of the WA-ZnO Nrods were controlled by varying the growth time from 1 h to 4 h. Finally, the WA-ZnO Nrods film was washed with DI water and dried at 120 °C for 20 min in air.

### 2.4. Device Fabrication

**Figure 1:**
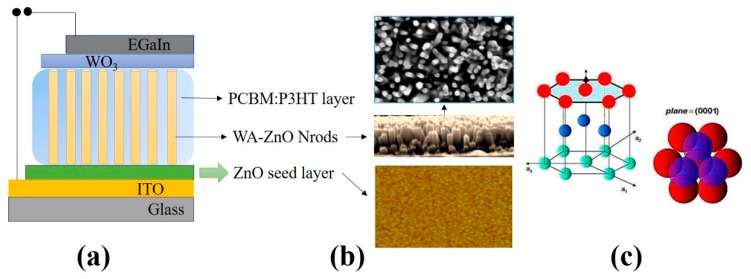
(**a**) The schematic diagram of the device structure, (**b**) WA-ZnO Nrods and ZnO seed layer morphology, and (**c**) WA-ZnO Nrod with hexagonal structure.

A mixture of P3HT:PCBM (1:0.8 in wt %) in chlorobenzene as a solvent was prepared and stirred overnight to form a composite solution. The active layer was deposited on the ITO/ZnO seed layer by spin-coating at 3000 rpm for 60 s and then dried at 120 °C for 10 min. The WO_3_ layer was then deposited by spin-coating at 4000 rpm for 40 s and dried at 120 °C for 10 min. Finally, E-GaIn (the cathode) was coated by brush-painting using a customized mask without using a vacuum process to complete the device structure of the glass/ITO/ZnO seed layer/WA-ZnO Nrods/(P3HT:PCBM)/WO_3_/E-GaIn. WO_3_ is effective for hole extraction in hybrid solar cells because of its good electron blocking capability, smooth morphology, and better ohmic contact between the photoactive layer and electrode [26,27]. In this study, WO_3_ was used as a hole transporting layer. Figure 1 shows a schematic diagram of the device structure, WA-ZnO Nrods and ZnO seed layer morphology, and WA-ZnO Nrod with hexagonal structure. 

### 2.5. Characterization

The surface and cross-section morphologies were determined by field emission scanning electron microscopy (FE-SEM, S-4800, Hitachi, Japan). The structures of the VA-ZnO Nrods were recorded by X-ray diffraction (XRD, PANalytical, X’Pert-PRO MPD, Malvern, UK) using Cu Kα radiation. The root mean square (RMS) roughness was measured by atomic force microscopy (AFM, Hitachi, Japan) and a video contact angle (VCA) technique. The current density–voltage (J–V) characteristics of the solar cells were measured using a solar simulator (Keithley 69911, Peccell Technologies, Inc., Yokohama, Japan) under AM 1.5 illumination.

## 3. Results and Discussion

The WA-ZnO Nrods were synthesized using a modified hydrothermal method [28,29,30], a ZnO seed layer was coated on the ITO/glass substrate by a spin coating method. The ZnO seed layer was annealed at 300 °C to improve adhesion on the substrate, and form a stable structure and good morphology, which help grow well-aligned ZnO Nrods [31].

The formation mechanism of the WA-ZnO Nrods can be explained by electron matching between the empty dangling bonds of the surface zinc atoms and the filled dangling bonds of the surface oxygen atoms with compensation of the internal dipolar moment of the wurtzite structure [32]. On the other hand, according to the theory of crystal nucleation and growth with the chemical process of the hydrothermal method, zinc nitrate hydrate and HMTA were used as the precursors to grow the ZnO Nrods. During the hydrothermal treatment, zinc nitrate hydrate provided the Zn^2+^ cations required for building up the WA-ZnO Nrods and HMTA hydrolysis supplied OH^−^ anions to form the WA-ZnO Nrods.

(CH_2_)_6_N_4_ + 6H_2_O → 4NH_3_ + 6HCHO (1)

NH_3_ + H_2_O → H^−^ + NH^4+^ (2)

Zn^2+^ + 2OH^−^→ Zn(OH)_2_ (3)

Zn(OH)_2_ → ZnO(s) + H_2_O (4)

The effects of the growth time on the structural and morphological properties of WA-ZnO Nrods were investigated by varying the growth time from 1 to 4 h.

**Figure 2:**
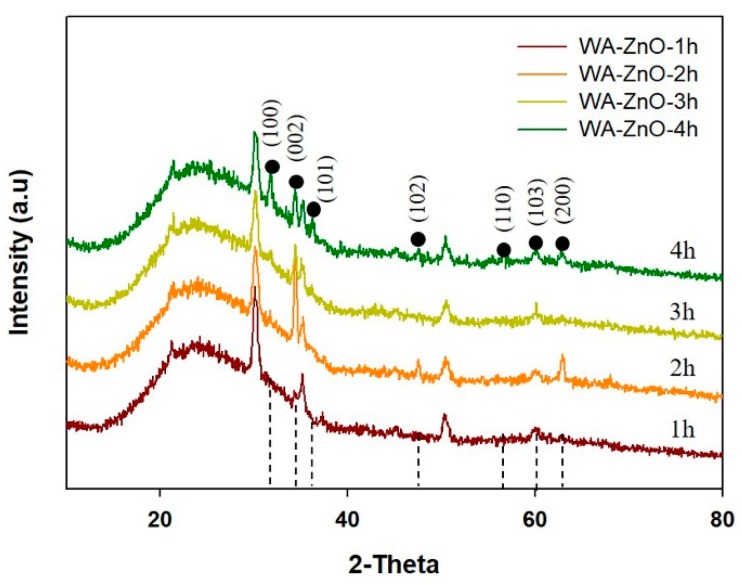
The X-ray diffraction pattern of WA-ZnO Nrods grown on the ITO substrate with various reaction time of 1 to 4 h.

Figure 2 presents XRD patterns of the WA-ZnO Nrods grown at different growth times. The patterns were indexed to the hexagonal wurtzite structure (Joint Committee on Powder Diffraction Standards) (JCPDS) Card No. 36-1451). During the growth process, the (002) peak initially appeared and the peak intensities increased with increasing reaction time, suggesting that the crystalline orientation following the peak (002) direction of the hexagonal structure was prioritized; the preferential peak (002) corresponds to the (0001) planes (referred to as (001) surface), which are parallel to the substrate surface. The orientation of the (0001) planes will allow preferred perpendicular growth of VA-ZnO Nrods on the substrate, coexisting with some off c-axis (100) and (101) [20].

At a 2 h growth time, the preferred orientation peak of (002) was strongest with a small contribution of other peaks, suggesting that the (002) plane is still the preferred orientation. When the reaction time was longer than 2 h, the intensity of the (002) peak was relatively unchanged and the intensities of the (100) and (101) peaks increased dramatically. This was explained by the different surface free energies associated with the different surface planes: <0001> (1.6 J/m^2^), <1120> (2.0 J/m^2^), and <1010> (3.4 J/m^2^). In the equilibrium state, the WA-ZnO Nrods grow in the plane with the lowest surface energy, so the WA-ZnO Nrods grow dominantly along the <0001> surface planes corresponding to the (002) peak [28,33,34]. After a 2 h reaction, the Zn^2+^ is depleted. The non-equilibrium state begins to form in the reaction and the reserve dissolution reaction according to Equation (3), which then becomes increasing competitive with the crystallization reaction until the equilibrium Zn^2+^ cation concentration is reached. Consequently, the other (100), (101), (102), and (103) peaks appear.

When the crystal growth of ZnO Nrods follow the (002) direction, the morphology of the ZnO Nrods are aligned vertically. With the appearance of various XED peaks correlated with ZnO Nrods, development followed in various directions with increasing reaction time. As a result, a confused surface with vertical, inclined, and horizontal ZnO Nrods appeared. 

**Figure 3:**
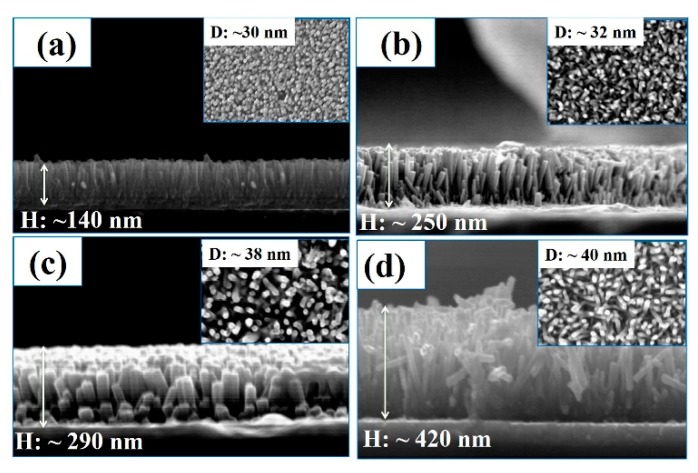
The scanning emission microscope of (top view (inset) and cross-section view) images of WA-ZnO Nrods grown on the ITO substrate with various reaction time of 1 to 4 h. (**a**) 1 h of growth time; (**b**) 2 h of growth time; (**c**) 3 h of growth time, (**d**) 4 h of growth time.

Figure 3a–d presents cross-section (FE-SEM) and top view (inset) images of ITO/WA-ZnO Nrods films with different of growth times of 1, 2, 3, and 4 h. The results showed that in all cases, the ZnO Nrods were well- aligned on the ITO substrate and corresponded to a peak orientation of (002). These results correspond to the above XRD study. The length (H) and diameter (D) of the ZnO Nrods were changed according to the varying growth times. When the growth time was increased from 1 to 4 h the length and diameter of the ZnO Nrods increased. XRD and FE-SEM showed that at a growth time of 2 h, the ZnO Nrods are vertically aligned with a high density compared to the other growth times.

**Figure 4:**
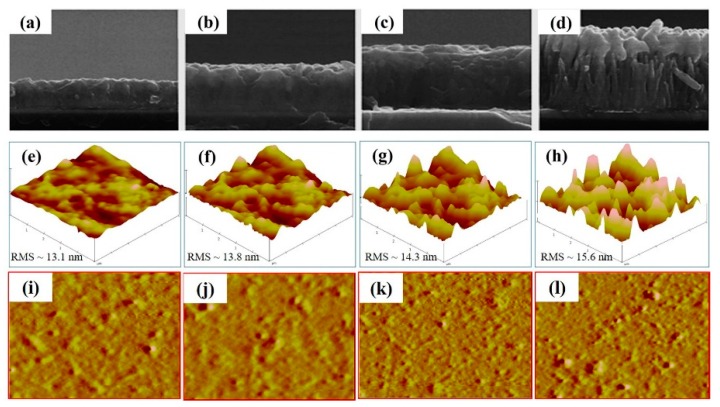
The SEM images of the (**a**–**d**) (WA-ZnO/P3HT:PCBM) active layer on ZnO seed layer/ITO/glass, 3D-AFM images of the (**e**–**h**) (WA-ZnO/P3HT:PCBM)- and 2D-AFM images of the (**i**–**l**) (WA-ZnO/P3HT:PCBM)-active layer on ZnO seed layer/ITO/glass with various reaction time of 1 to 4 h

The WA-ZnO Nrods have been applied widely in inverted organic hybrid solar cells [15]. The device performance improved with an increasing average length of the ZnO Nrods due to the improved carrier transport pathway to the cathode in the devices. In the present study, synthesized- WA-ZnO Nrods with different lengths were applied to hybrid photovoltaic as a carrier transport layer. Figure 4 presents the typical morphologies of WA-ZnO/P3HT:PCBM films after intercalation with P3HT:PCBM. Figure 4a–d show cross-sectional FE-SEM images of WA-ZnO/P3HT:PCBM films. The P3HT:PCBM effectively filled the interspaces between the nanorods.

To produce a better blend morphology between the composite solution and VA-ZnO Nrod structure, the structure requires a long rod length, while also maintaining space for infiltration of the composite solution. Composite solution infiltration into the valleys of the VA-ZnO Nrods 1, 2 and 3 h samples were improved when growth times were increased as shown in (Figure 4a–d). Therefore, excitons can easily move along the path because of the enhanced interfacial contact between the organic and inorganic materials in OPVs. The device structure of the ZnO Nrods (4 h reaction time) had a low V_OC_ and J_SC_ because the rod length was so high and the structure contained inclined and horizontal nanorods, which impeded the movement of polymer to the rod valley.

**Figure 5:**
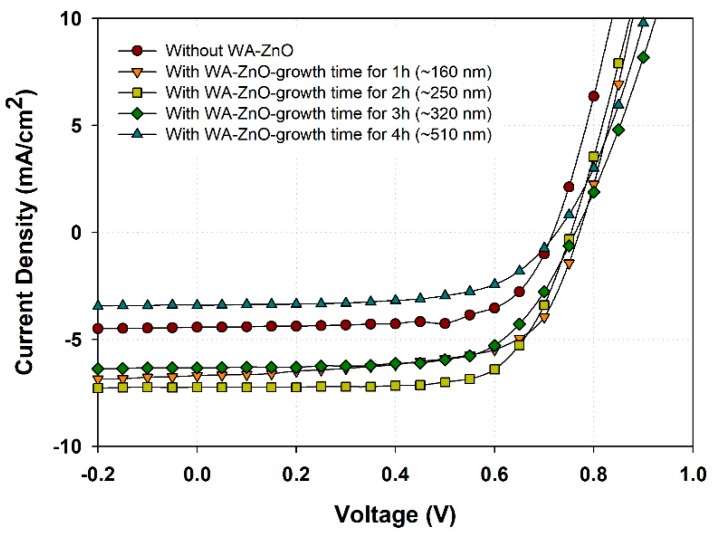
J–V curve characteristics of the device with structure of glass/ITO/ZnO seed layer/WA-ZnONrod/(P3HT:PCBM)/WO_3_/EGaIn with various reaction time of 1 to 4 h.

The surface roughness (RMS) of the films prepared from WA-ZnO Nrods with different growth times of 1, 2, 3, and 4 h were 13.1, 13.8, 14.3, and 15.6 nm, respectively. These small variations in surface roughness are due to the different densities and vertically aligned ZnO Nrods. High-density and uniform vertically aligned nanorods are expected to offer a large ZnO/active layer interface and improve the glass/ITO/ZnO seed layer/WA-ZnO Nrods/(P3HT:PCBM) surface morphology. We fabricated a device with a structure of ITO/WA-ZnO Nrods/(P3HT:PCBM)/WO_3_/EGaIn and we measured the short-circuit current density (J_SC_), open-circuit voltage (V_OC_), fill factor (FF), and power conversion efficiency (PCE), as shown in Figure 5 and Table 1. Figure 5 shows that at 2 h growth time, the device had a maximum efficiency (3.8%, 6.78 mA/cm^2^) with a ZnO Nrod length of ~250 nm (shown in Figure 3b). The J_SC_ of the device (1 h and 2 h of reaction time) was higher than the others (Table 1). This suggests that the electron transportation ability of the ZnO Nrods containing only vertically aligned nanorods is better than the structure with inclined or horizontal nanorods. 

**Figure 6:**
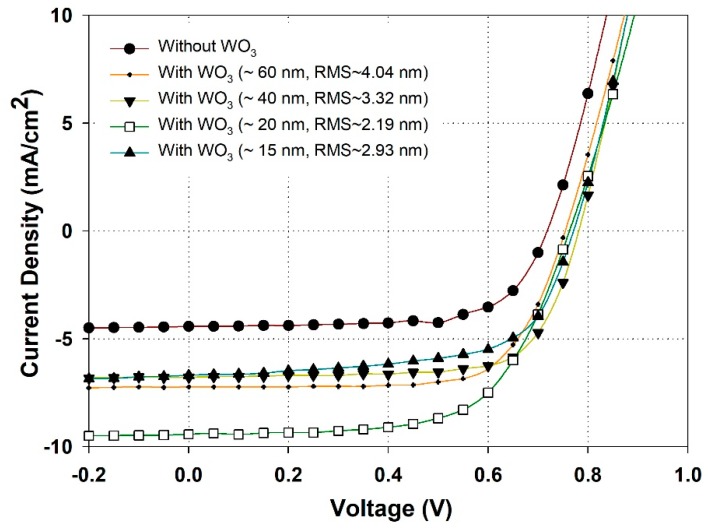
J–V curve characteristics of the device with structure of glass/ITO/WA-ZnONrod (250 nm)/(P3HT:PCBM)/WO_3_/EGaIn dependent on the thickness and morphology of WO_3_ buffer layer.

The effects of thickness and morphology of WO_3_ buffer layer on the device performance was studied by varied the spin speed in range between 4000 and 6000 round per minute (rpm). Figure 6 shows the J–V curves of the device without- and with- WO_3_ layer with different thickness and surface roughness. In the case of without WO_3_, the device with structure of glass/ITO/WA-ZnONrod/(P3HT:PCBM)/EGaIn shows a PCE of 2.1% with a very low current density of 4.40 mA/cm^2^. The FF and V_oc_ are 67.1% and 0.725 V, respectively. However, by adding a 60 nm WO_3_ layer between photoactive layer (P3HT:PCBM) and EGaIn, the device exhibits a PCE of 3.8% with J_sc_ = 7.17 mA/cm^2^, V_oc_ = 0.75 V, and FF = 71.0%. By inserting a 60 nm WO_3_ layer, the current density is increased from 4.40 to 7.17 mA/cm^2^, an improvement of 63%. Our result shows that WO_3_ effectively reduces the recombination of the charge carriers and enhances hole collection efficiency at the photoactive layer/EGaIn interface [35]. The WO_3_ layer needs to cover the photoactive layer and EGaIn electrode fully and uniformly to improve the leakage current, so the optimization of the buffer layer thickness and surface morphology was studied, as shown in Figure 6 and Table 2. When the thickness of buffer layers was varied from 60 nm to 15 nm, the device performance was improved. Specifically, the maximum power conversion efficiency is about ~4.5% (with J_sc_ = 9.1 mA/cm^2^, V_oc_ = 0.77 V, and FF = 71.3%). Our results show that the optimum thickness for WO_3_ layer is about ~20 nm, thinner values lead to poor coverage of the film, while thicker values would increase the resistance, thus, lowering device performance.

**Figure 7:**
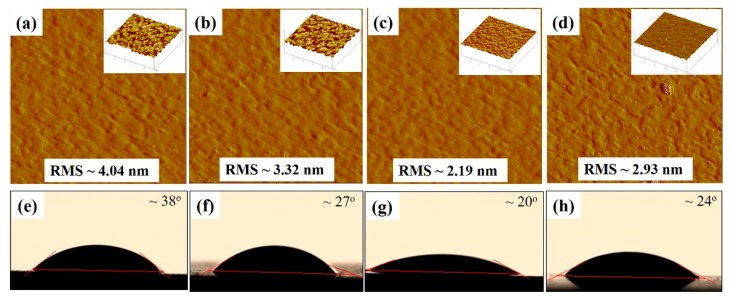
The AFM phase images of the (**a**–**d**) glass/ITO/WA-ZnONrod/(P3HT:PCBM)/WO_3_, (inset) AFM 3-Dimention images, and video contact angle images of the (**e**–**h**) glass/ITO/WA-ZnONrod/(P3HT:PCBM)/WO_3_ dependent on the thickness and morphology of WO_3_ buffer layer.

The glass/ITO/WA-ZnONrod/(P3HT:PCBM)/WO_3_ surface morphology with different WO_3_ thickness was studied by AFM and VCA technique, as shown in Figure 7.

Decreasing the thickness of the WO_3_ layer by increasing the spin speed led to a decrease in the roughness of glass/ITO/WA-ZnONrod/(P3HT:PCBM)/WO_3_. The best device performance corresponds to 20 nm of thickness of the WO_3_ layer and 2.19 nm of roughness value (contact angle is about ~20°). Moreover, the surface roughness of WO_3_ thin films, which contributes to the surface energy can affect the properties of interfacial of the WO_3_/active layer. Figure 7e–h shows the measured contact angle of glass/ITO/WA-ZnONrod/(P3HT:PCBM)/WO_3_ with different surface roughness. The contact angle decreases from 38 to 20° when the surface roughness decreases from 4.04 to 2.19 nm and increases again from 20 to 24° when roughness increases from 2.19 to 2.93 nm. This result indicates that the surface morphology of glass/ITO/WA-ZnONrod/(P3HT:PCBM)/WO_3_ of different WO_3_ surface roughness should be different.

When the thickness of WO_3_ is 15 nm or less, the photoactive layer is not completely covered by WO_3_, leading to small WO_3_ islands forming on the sample surface thus increasing the surface roughness, as shows in Figure 7d. Increased surface roughness results in poor device performance because an increase in the contact resistance between active layer/electrode. The results showed that in all cases, inserting a WO_3_ into a device as a HTL (20 nm optimum thickness) improved performance compared with a device without a WO_3_ HTL, because lower surface roughness leads to less electron blocking and better hole collection efficiency at the photoactive/EGaIn.

## 4. Conclusions

In summary, we have investigated the application of synthesized-WA-ZnO Nrods and WO_3_ as a carrier transport layer in (P3HT:PCBM) vacuum free hybrid photovoltaic. We fabricated a device structure of glass/ITO/WA-ZnONrod (~250 nm)/(P3HT:PCBM)/WO_3_(~20 nm)/EGaIn, and we measured a maximum power conversion efficiency of ~4.5% (J_sc_ = 9.1 mA/cm^2^, V_oc_ = 0.77 V, and FF = 71.3%). This improves upon previous results with power conversion efficiency of ~3%. We attribute the improved device performance to smoother surface roughness at the active layer/EGaIn interface, better electron blocking, and better hole/electron collection efficiency to the electrode. Further optimization of this device by changing the absorption polymer and nanostructure of the device is currently underway.

## Figures and Tables

**Table 1:** Device parameters without- and with VA-ZnO Nrod (varied of length) such as short-circuit current density (J_SC_), open-circuit voltage (V_OC_), fill factor (FF), and power conversion efficiency (PCE) were compared.

Sample	V_oc_ (V)	J_sc_ (mA cm^−2^)	FF (%)	PCE (%)
Cell-without WA-ZnO Nrods	0.725	4.42	67.1	2.1
Cell-with WA-ZnO Nrods (~160 nm)	0.750	6.60	63.7	3.3
Cell-with WA-ZnO Nrods (~250 nm)	0.750	6.78	71.0	3.8
Cell-with WA-ZnO Nrods (~320 nm)	0.780	6.30	65.7	3.1
Cell-with WA-ZnO Nrods (~510 nm)	0.725	3.41	61.8	1.5

**Table 2:** Device parameters such as short-circuit current density (J_SC_), open-circuit voltage (V_OC_), fill factor (FF), and power conversion efficiency (PCE) of device dependent on the thickness and morphology of WO_3_ buffer layer.

Sample	V_oc_ (V)	J_sc_ (mA cm^−2^)	FF (%)	PCE (%)
Cell-without WO_3_	0.725	4.4	67.1	2.1
Cell-with WO_3_ (~60 nm, RMS~4.04 nm)	0.750	6.78	71.0	3.8
Cell-with WO_3_ (~40 nm, RMS~3.32 nm)	0.750	7.17	71.0	3.9
Cell-with WO_3_ (~20 nm, RMS~2.19 nm)	0.770	9.40	62.6	4.5
Cell-with WO_3_ (~15 nm, RMS~2.93 nm)	0.750	6.69	63.0	3.2
